# Transcriptomic Analysis of HPV-Positive Oesophageal Tissue Reveals Upregulation of Genes Linked to Cell Cycle and DNA Replication

**DOI:** 10.3390/ijms26010056

**Published:** 2024-12-24

**Authors:** Muhammad Osama Shafiq, Muharrem Okan Cakir, Ugur Bilge, Yasmin Pasha, G. Hossein Ashrafi

**Affiliations:** 1 School of Life Sciences, Pharmacy and Chemistry, Kingston University London, London KT1 2EE, UK; 2 Department of Biostatistics and Medical Informatics, Faculty of Medicine, Akdeniz University, Antalya 07050, Turkey; 3 Department of Gastroenterology, Kingston Hospital, Kingston Upon Thames, London KT2 7QB, UK

**Keywords:** human papillomavirus (HPV), HPV positive oesophageal tissue, non-cancerous oesophageal tissue, transcriptomic profiling, cell cycle dysregulation, DNA replication, polymerase chain reaction (PCR), RNA sequencing

## Abstract

Human papillomavirus (HPV) is a prevalent sexually transmitted infection, implicated in various cancers, yet its influence in non-cancerous oesophageal tissue remains unclear. This study aims to investigate the gene expression changes associated with high-risk HPV (HR-HPV) in non-cancerous oesophageal tissue to elucidate potential early oncogenic mechanisms. Using RNA sequencing, we compared transcriptomic profiles of HPV-positive and HPV-negative non-cancerous oesophageal tissues. Differential gene expression analysis revealed significant upregulation of cell cycle and DNA replication pathways in HPV-positive samples, specifically involving key genes such as *CCNA2*, *DSN1*, and *MCM10*, which are known to regulate cellular proliferation and genomic stability. Additionally, kinase and transcription factor enrichment analyses highlighted HR-HPV-associated regulatory molecules, including E2F4 and CSNK2A1, suggesting HPV’s role in modulating host cell cycle control. These findings support the hypothesis that HPV infection may initiate cellular alterations in oesophageal tissue, potentially predisposing it to malignancy. This study contributes to understanding HPV’s impact in non-cancerous tissues and identifies possible biomarkers for early HPV-related cellular changes, offering insights into HPV-driven cancer development beyond traditionally associated sites.

## 1. Introduction

Human papillomaviruses (HPVs) are small, circular, double-stranded DNA viruses, approximately 55 nm in diameter, with a non-enveloped icosahedral capsid structure [1]. As the most prevalent sexually transmitted infections (STIs) worldwide, HPVs infect a significant portion of the global population, resulting in a wide range of health conditions [2]. Over 200 HPV types have been identified and categorised based on their oncogenic potential into two main groups: high-risk HPVs (HR-HPVs) and low-risk HPVs (LR-HPVs). While LR-HPVs are primarily associated with benign conditions such as cutaneous and genital warts, HR-HPVs are strongly linked to several cancers, including those of the cervix, anus, oropharynx, vulva, penis, and skin, as well as precursor lesions associated with these cancers [2,3,4].

HR-HPVs encode three principal oncoproteins, E5, E6, and E7, which play essential roles in cellular transformation and cancer progression. The E5 protein, for instance, interacts with major histocompatibility complex (MHC) class I molecules, retaining them within the Golgi apparatus and thereby preventing their transport to the cell surface. This action helps the virus evade immune surveillance, supporting persistent infection [5,6]. Additionally, the E6 protein promotes cellular immortalisation by binding to the tumour suppressor protein p53, leading to its ubiquitination and degradation, which interferes with the normal apoptotic response. Similarly, the E7 protein fosters uncontrolled cell proliferation by inactivating the RB protein, thus releasing E2F transcription factors that drive DNA synthesis and cell division [7].

Although the presence and role of HR-HPVs in various cancers are well established, their presence in non-cancerous tissue is often overlooked. Investigating HPV infection in tissues where malignancy is not yet developed could provide vital insights into early viral activity and its potential role in cancer initiation. Understanding HPV interaction with host cells and identifying altered gene expression or protein profile in non-cancerous tissue may reveal mechanisms that contribute to cellular transformation and identify biomarkers associated with early HPV infection, which could aid in developing diagnostic tools for early detection.

The presence of HPV in the oesophagus and its potential implications are crucial, though not well understood. While extensive research has established a clear association between HPV and various cancers, the role of HPV infection in the oesophagus is not conclusive in terms of cancer and is especially understudied in non-cancerous tissue. In this study, we addressed this gap by evaluating the differential gene expression in HPV-positive oesophageal samples and examining how these changes impact regulatory molecules. Our findings revealed an upregulated expression of key genes involved in cell proliferation, which may drive infected tissue toward pathological changes.

## 2. Results

### 2.1. Pathological Characteristics and Detection of HR-HPV

The histopathological evaluation of the samples was carried out by the pathology department of Kingston Hospital and all six samples were reported to be non-cancerous inflamed tissue. Tissue specimens from the fresh non-cancerous tissues were examined for the presence of DNA of 14 HR-HPV types using type-specific real-time PCR.

The data obtained revealed that HR-HPV DNA was present in three non-cancerous oesophageal tissue samples tested. All the positive samples had HR-HPV 56 infection. One of the three HPV-positive samples had an additional infection of HR-HPV 18 showing co-infection with HR-HPV 56. The remaining three samples were HR-HPV infection-free. The amplification of the *β-globin* gene was positive in all extracted DNA, indicating an adequate quality of DNA. Positive and negative controls confirmed that there was no evidence of contamination, indicating a successful PCR amplification.

### 2.2. Transcriptomic Profiling of Non-Cancerous Oesophageal Tissue with and Without HR-HPV Infection

**Figure 1:**
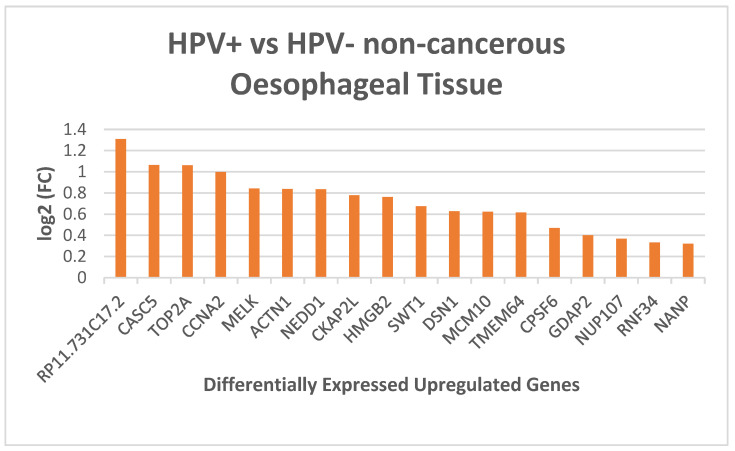
Differentially expressed genes between HPV-positive and HPV-negative non-cancerous human oesophageal tissue samples *p*-value (*p* < 0.001).

To investigate the impact of HPV on the gene expression profile of oesophageal tissue, we performed RNA sequencing (RNA-Seq) to analyse changes in gene expression across three HPV-positive and three HPV-negative oesophageal samples. The subsequent analysis of differential gene expression revealed that 18 genes were consistently and significantly upregulated in HPV-positive samples compared to HPV-negative non-cancerous oesophageal human tissue samples (Figure 1). A description of genes function is provided in the Supplementary Materials File S1 [8]. Gene expression values for HPV-positive and HPV-negative non-cancerous oesophageal samples, along with statistical significance (*p*-values), are detailed in Supplementary Material File S2.

### 2.3. Analysis of Differentially Expressed Genes of Non-Cancerous Oesophageal Tissue with and Without HR-HPV Infection Using Pathway Enrichment Analysis

Differentially upregulated genes in HPV-positive oesophageal tissue were analysed using pathway enrichment analysis. The results showed that the genes significantly upregulated following HPV infection are involved in cell proliferation including pathways of “cell cycle” and “DNA replication” (Table 1). Notably, some genes are overlapping between the pathways, indicating their central role in cell proliferation.

### 2.4. Investigation of Differentially Expressed Genes of Non-Cancerous Oesophageal Tissue with and Without HR-HPV Infection via Kinase Enrichment Analysis

**Figure 2:**
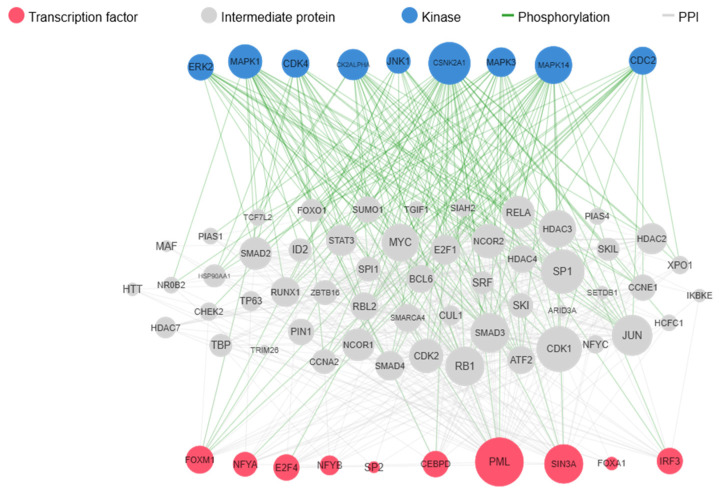
Complete upstream pathways connecting enriched transcription factors to kinases through known protein-protein interaction. Red nodes highlight top transcription factors predicted to regulate our upregulated genes. Grey nodes depict proteins that interact with these transcription factors. Blue nodes represent kinases predicted to phosphorylate within the network. Green lines represent phosphorylation interactions between kinases and their targets, while grey lines indicate protein-protein physical interactions. Adapted with permission from Ref [9]. Copyright 2012, Ma’ayan Lab.

To identify molecular networks modulated by HR-HPV infection, we used kinase enrichment analysis (KEA) to examine key kinases and transcription factors that regulate the differentially expressed gene expression between HPV-positive and HPV-negative groups. The results highlighted the prominence of transcription factors including E2F4, FOXM1, CEBPD, IRF3, and NFYA along with kinases CSNK2A1, CDK4, CDK2ALPHA, MAPK14, and CDK1 (Table 2A,B). These molecules play an important role in altering the expression of observed genes indicating a complex interaction between HPV and cellular regulatory network. The upstream pathway analysis of upregulated transcription factors and kinases indicated that HPV infection influences key regulatory molecules involved in the cell cycle including CDK1, RB1, CDK2, and SP1 (Figure 2). The observation of these transcription factors and kinases as the key regulators of upregulated genes in HPV-positive non-cancerous samples points to the pathways that HPV might influence to alter cellular functions, even in non-cancerous states.

## 3. Discussion

Human papillomavirus (HPV), the most prevalent sexually transmitted infection globally, affects millions and contributes to a range of diseases from benign warts to cancers across multiple sites [2,3,4,10]. High-risk HPV (HR-HPV) types are implicated in approximately 750,000 cancer cases worldwide each year [11]. While HPV’s role in cancers of the cervix, head, and neck is well documented, its potential effects on the oesophageal tissue remain poorly understood, particularly in non-cancerous tissue. HPV infection in the oesophagus was first proposed in the early 1980s [12], but despite numerous studies, its connection to oesophageal cancer remains inconclusive, complicated by variations in geography, HPV type, and detection methods [13].

Given the complexity of cancer biology, investigating HPV’s effects on the host genome in non-cancerous tissues offers unique insights, as cancer-associated genomic changes can arise independently of HPV infection. Our study explores whether the presence and activity of HPV in non-cancerous oesophageal tissues may alter the host genome, potentially initiating a progression toward oesophageal cancer driven solely by HPV expression. All the positive samples had HR-HPV 56 infection. One of the three HPV-positive samples had an additional infection of HR-HPV 18, showing co-infection with HR-HPV 56. HPV 56 has been reported in cervical cancers, accounting for less than 1% of cervical cancer cases worldwide [14]. It has also been detected in some oesophageal cancer cases, although the literature on HPV 56 remains limited. An increase in the distribution of HPV 56 has been observed recently, potentially due to exclusion from HPV vaccination coverage [15,16]. Its detection in this study underscores its potential relevance to oesophageal tissue, highlighting the need for further investigation into its biological significance beyond the cervix, where it has been mostly reported.

Using RNA-Seq analysis, we compared the transcriptome of HPV-positive non-cancerous oesophageal tissues to HPV-negative controls, identifying significant alterations in gene expression linked to the cell cycle (*p*-value: 7.49 × 10⁻⁸) and DNA replication pathways (*p*-value: 8.69 × 10⁻⁵). The upregulation of cell cycle regulation pathways in HPV-positive, non-cancerous oesophageal tissues may cause abnormal cell proliferation, a hallmark of carcinogenesis [17].

Key cell cycle regulatory genes, including *CCNA2*, *NEDD1*, and *DSN1*, were significantly overexpressed in these samples. For instance, *CCNA2* is pivotal in cell cycle control, with dysregulation linked to tumour development [18]. Likewise, *NEDD1* and *DSN1*, crucial for microtubule organisation and chromosome segregation during cell division, are linked to genomic instability and abnormal cell division when overexpressed [19,20,21]. The dysregulation of cell cycle pathways in our findings aligns with established mechanisms in HPV-related cancers, such as cervical cancers and head and neck cancers, where HPV oncoproteins E6 and E7 disrupt cellular controls, inducing genomic instability and cell cycle dysregulation [22,23].

Additionally, our results indicate a significant upregulation in DNA replication pathways, with key genes such as *CASC5* and *MCM10* overexpressed in HPV-positive samples. *MCM10*, part of the mini-chromosome maintenance complex, is essential for DNA replication and genomic stability, and its aberrant expression is implicated in various cancers, including cervical cancer [24,25]. Similarly, *CASC5*, essential for chromosome segregation during cell division, is associated with cancer when dysregulated [26,27]. This upregulation suggests that HPV infection may influence DNA replication, potentially increasing genomic instability in non-cancerous tissue both of which are considered key contributors to the hallmarks of cancers [28].

The upregulation of genes in the cell cycle and DNA replication pathways correlates with the documented mechanisms of HPV E6 and E7 oncoproteins. E7 facilitates unchecked cell proliferation by inhibiting the retinoblastoma protein (pRB), allowing the premature entry of cells into the S-phase [29]. Additionally, E6 promotes the degradation of the p53 tumour suppressor, which usually pauses cell division in response to DNA damage, preventing apoptosis. The degradation of p53 permits cells to accumulate genetic mutations, contributing to genomic instability and malignancy risk [30]. These established mechanisms in cervical and head and neck cancers suggest that similar pathways may be disrupted by HPV in non-cancerous oesophageal tissue, contributing to cell cycle and DNA replication disturbances.

Longitudinal studies have shown that persistent HR-HPV infection in normal tissues of the cervix and the head and neck can lead to cancer over time [31,32,33]. HR-HPV is central to the transformation of normal cervical tissue to carcinoma, primarily through cell cycle dysregulation and genomic instability. Similarly, studies on HPV infection in normal head and neck tissues indicate that while most infections are transient, persistent infection can lead to oral and oropharyngeal cancers [34,35]. Our findings align with this pattern, suggesting that HPV infection in non-cancerous oesophageal tissue may similarly initiate cellular changes that could drive cancer development over time.

To further investigate molecular networks modulated by HPV, transcription factor and kinase enrichment analysis was performed, revealing the involvement of transcription factors such as E2F4, FOXM1, and kinases CSNK2A1 and CDK4. These transcription factors are integral to cycle regulation, differentiation, and DNA synthesis. Elevated expressions of these transcription factors are associated with tumorigenesis in a variety of cancers [36,37,38,39,40,41,42,43]. CSNK2A1, a serine/threonine kinase is a catalytic subunit of Casein Kinase II (CK2), involved in various cellular processes including cell cycle regulation, apoptosis, signalling pathways, growth, and metabolism [44]. Overexpression of CSNK2A1 is reported to play an important role in various cancers including gastric cancer and breast cancer [45,46]. CDK4, a serine/threonine kinase, is associated with cell cycle regulation, especially the transition from the G1 to the S phase. CDK4 pairs with D-type cyclins and drives cell cycle progression, thereby promoting proliferation [47]. Overexpression of CDK4 has been observed in a variety of cancers including breast cancers, glioblastomas multiforme, gliomas, and meningiomas [48]. Our pathway analysis reveals the impact of HR-HPV infection on essential proteins involved in the cell cycle and DNA replication pathways including SP1, E2F1, CDK1, and CDK2 [49,50]. The identification of these kinases and transcription factors points to a complex regulatory network that may be disrupted by HPV infection, further supporting the virus’s role in creating a pro-proliferative environment conducive to oncogenic transformation.

Despite the small sample size, this study provides important preliminary insights into gene expression differences between HPV-positive and HPV-negative non-cancerous oesophageal tissues. Similar or smaller sample sizes have been used in previous RNA sequencing studies, demonstrating that meaningful biological conclusions can still be drawn using robust bioinformatics tools such as DESeq2 [51,52]. Due to the anonymised nature of sample collection, detailed demographic and clinical data were unavailable, limiting the exploration of patient-specific factors. The genes highlighted in our study are known to play roles in cell cycle regulation and DNA replication pathways. While these pathways may be activated by various stimuli, their consistent upregulation in HPV-positive samples supports HPV’s influence. Future research should consider larger cohorts, incorporate additional clinical information, and apply multi-omics approaches to validate the findings and assess the potential of identified genes as biomarkers.

In conclusion, our study suggests that HR-HPV infection in non-cancerous oesophageal tissue leads to cell cycle dysregulation and may encourage precancerous changes through genomic instability. The upregulation of key genes and regulators, such as FOXM1, CSNK2A1, and CDK4, highlights potential biomarkers for early detection of HPV-related cellular changes. These findings emphasise the significance of HPV’s impact on the oesophagus, suggesting that even in non-cancerous tissue, HR-HPV may exert oncogenic pressure and predispose cells to malignancy. To our knowledge, this is the first study to compare gene expression in HPV-negative and HPV-positive non-cancerous oesophageal tissue, providing insights into the molecular alterations induced by early HPV infection.

## 4. Materials and Methods

### 4.1. Oesophageal Tissue Specimen Collection

The study was conducted with the approval of the Health Research Authority (HRA) and Health and Care Research Wales (HCRW) under project ID 250010. All procedures strictly adhered to approved guidelines and regulations. Informed consent was obtained from the patients participating in the study. The study protocol included the protection of patient-identifiable information from the research team.

Patients referred to Kingston Hospital, London by general practitioners (GPs) because of gastrointestinal complaints gave consent, and oesophageal biopsies (3–6 per patient) were collected by the endoscopy team in the endoscopy unit of the Kingston Hospital. The diagnosis of all oesophageal tissue specimens was formally reported by the Kingston Hospital pathology department as part of the standard clinical diagnosis of patients.

Following excision, the integrity of the biopsy tissues was immediately preserved by submerging them in the ALLPROTECT reagent (QIAGEN, Hilden, Germany). This step aimed to stabilise DNA, RNA, and protein while maintaining aseptic handling to prevent contamination of samples. The specimens were transferred to Kingston University in accordance with World Health Organisation transport guidelines for infectious material [53].

### 4.2. Genomic Material Extraction and Purification

To avoid cross-contamination between specimens, separate disposable items such as gloves, petri dishes, tubes, and surgical blades, were used for the handling of each specimen. Cellular DNA, RNA, and protein were extracted from the oesophageal specimens using a column-based extraction kit. The samples were fragmented and homogenised in lysis buffer using the Tissue Lyser (QIAGEN, Hilden, Germany) and further processed with the QIAshredder spin column (QIAGEN, Hilden, Germany).

The extraction of genomic material and protein was conducted using GenElute RNA/DNA/Protein Plus Purification Kit (Sigma-Aldrich, St. Louis, MO, USA) according to the manufacturer’s protocol. Purification of these biological molecules in this kit is based on spin column chromatography allowing the sequential isolation of genomic DNA, total RNA, and protein from a single sample. The concentration and purity of eluted genomic material were assessed using the spectrophotometer BIODROP DUO+ (Biochrom, Cambridge, UK).

### 4.3. Detection and Genotyping of HPV DNA

The methodology employed for the detection and identification of HPVs involved the utilisation of real-time polymerisation chain reaction (PCR). The AmpliSens HPV HCR genotype-Titre-FRT PCR kit (Ecoli s.r.o., Bratislava, Slovak Republic) was used according to the manufacturer’s direction for the detection and genotyping of 14 high-risk HPV types including types 16, 18, 31, 33, 35, 39, 45, 51, 52, 56, 58, 59, 66, and 68 in 52 oesophageal tissue specimens, extracted at Kingston University laboratories, UK. The presence of HPV was detected using Stratagene Mx3005p (Agilent, Santa Clara, CA, USA). Each PCR run contained the quality control measures. This included positive control, negative control, and internal control (*β-globin*) primers. These measures were integral to mitigating the risk of false positive or false negative results. To avoid cross-contamination, the DNA extraction and PCR amplification were carried out in separate laboratories following strict aseptic protocol.

### 4.4. RNA Sequencing

The RNA sequencing was performed on six non-cancerous oesophageal tissue samples, divided into two groups: an HPV-positive group and an HPV-negative group, with three samples in each. All six samples had RIN values greater than 7.5. The library preparation, sequencing and bioinformatics analysis were performed by CeGaT GmbH, Tubingen, Germany. The SMART-Seq standard kit (Takara, Kusatsu, Japan) was utilised for library preparation and multiplexed libraries were sequenced using the Illumina NovaSeq 6000 platform (Illumina, San Diego, CA, USA) at 100 bp paired-end reads. The sequencing depth for each sample was >20 million reads. All samples passed quality control based on the manufacturer’s standards.

### 4.5. Bioinformatics Analysis

Diverse bioinformatics tools were utilised for the analysis of sequence reads. The sequence reads were de-multiplexed with Illumina bcl2fastq (vs. 2.20; Illumina Inc, San Diego, CA, USA) and adaptors were trimmed using Skewer (vs. 0.2.2; Hong Kong Baptist University, Hong Kong, China) [54]. STAR (vs. 2.7.3; Alexander Dobin, Cold Spring Harbor Laboratory, Cold Spring Harbor, NY, USA) was utilised for the alignment of trimmed raw reads to hg19-cegat [55]. Pseudo-autosomal regions (PAR) on the Y chromosome were masked as reads from these regions and were also mapped to their corresponding locations on the X chromosome. Normalised counts were calculated using DESeq2 (version 1.24.0; Michael I. Love, T.H. Chan school of Public Health, Boston, MA, USA) in R (version 3.6.1; R Foundation for Statistical Computing, Vienna, Austria) [56]. For the functional enrichment analysis, the RNA-Seq data obtained from HPV-positive and HPV-negative oesophageal tissue were used. Gene set enrichment analysis (GSEA) was performed using the GSEA software (version number: 4.3.0; Broad Institute, Cambridge, MA, USA) [57,58]. The RNA-Seq data sets were pre-processed and normalised, and the resulting gene expression profiles were analysed against a comprehensive collection of gene sets derived from public databases, such as MSigDB [58]. The GSEA algorithm computed an enrichment score for each gene set, indicating the extent to which the gene set was overrepresented among the differentially expressed genes.

EnrichR, an online platform, was used for comprehensive gene set enrichment analysis (Online platform, Ma’ayan Laboratory, Icahn School of Medicine at Mount Sinai, New York, NY, USA) [59,60,61]. The pre-processed RNA-Seq data sets were uploaded to EnrichR and analysed following the provided instructions. EnrichR identifies pathways associated with differentially expressed genes. The analysis yielded enriched pathways results along with corresponding statistical significance. Outputs from GSEA and EnrichR were utilised to understand the biological processes and pathways impacted by the infection of HR-HPVs in human oesophageal tissue. The kinase enrichment analysis was confirmed using Expression2 Kinase (X2K) software (vs 0.0.4, Ma’ayan Laboratory, Icahn School of Medicine at Mount Sinai, New York, USA) [9,62].

## Figures and Tables

**Table 1:** Pathway enrichment analysis of upregulated genes in HPV-positive oesophageal tissue. The table includes the description of pathways, the number of overlapping genes from the differentially overexpressed genes, the *p*-value, and the overlapping genes.

Description	Number of Overlapping Genes	*p*-Value	Overlapping Genes
Cell Cycle	7	7.49 × 10^−8^	*TOP2A*; *CCNA2*; *NEDD1*; *DSN1*; *NUP107*; *CASC5*; *MCM10*
DNA Replication	5	8.69 × 10^−5^	*CCNA2*; *DSN1*; *NUP107*; *CASC5*; *MCM10*

**Table 2:** Analysis of transcription factors and kinases regulating gene upregulation upon HPV infection. (A) Top transcription factors involved. (B) The top regulatory kinases.

(**A**)	
**Transcription Factors**	***p*-Value**
E2F4	1.6 × 10^−8^
FOXM1	1.5 × 10^−4^
CEBPD	9.1 × 10^−4^
(**B**)	
**Regulatory Kinases**	***p*-Value**
CSNK2A1	2.2 × 10^−15^
CDK4	7.0 × 10^−15^
MAPK14	1.05 × 10^−12^

## Data Availability

Data are available upon request from the authors.

## Supplementary Materials

The supporting information can be downloaded at: https://www.mdpi.com/article/10.3390/ijms26010056/s1.
