# Supplementary material for: Transcriptomic Analysis of HPV-Positive Oesophageal Tissue Reveals Upregulation of Genes Linked to Cell Cycle and DNA Replication
Source: Int J Mol Sci. 2024 Dec 24;26(1):56. doi: 10.3390/ijms26010056 (PMC11720088; doi:10.3390/ijms26010056)
Supplement: Supplementary file 1 [file ijms-26-00056-s001.zip › Supplementary Material File S1.pdf]

Table S1. A short description of statistically significant ($p < 0.001$)) upregulated genes in HR-HPV positive non-cancerous oesophageal samples [1].

Gene	Function
RP11-731C17.2	Non-coding
CASC5	A key player in chromosome segregation and mitotic spindle checkpoint regulation
TOP2A	Encodes DNA topoisomerase II, essential for DNA replication, transcription, and cell division
CCNA2	Cyclin A2, regulates the cell cycle by controlling the transition from G1/S and G2/M phases
MELK	Involved in cell cycle regulation, stem cell maintenance, and oncogenesis
ACTN1	Encodes actinin, involved in cytoskeletal organization and cellular adhesion
NEDD1	Participates in centrosome assembly and microtubule organization
CKAP2L	Critical for mitotic spindle stability and chromosome segregation
HMGB2	A chromatin-binding protein involved in DNA repair and transcription regulation
SWT1	Linked to RNA splicing and cellular response to DNA damage
DSN1	A component of the kinetochore involved in chromosome alignment during cell division
MCM10	Essential for the initiation of DNA replication and chromatin binding
TMEM64	Implicated in bone metabolism and immune cell regulation
CPSF6	Regulates mRNA processing and 3'-end cleavage of pre-mRNAs
GDAP2	Linked to mitochondrial dynamics and cellular stress responses
NUP107	Component of the nuclear pore complex, facilitating nucleocytoplasmic transport
RNF34	A ubiquitin ligase involved in protein degradation and immune signalling
NANP	A gene associated with neurodevelopment and cell signalling

Reference:

1. National Center for Biotechnology Information (NCBI)[Internet]. Bethesda (MD): National Library of Medicine (US), National Center for Biotechnology Information; [1988] – [Cited 2024 Dec 12]. Available from: Https://Www.Ncbi.Nlm.Nih.Gov/.
